# Indocyanine Green Retention Rate at 15 Minutes as a Key Predictor of Clinically Significant Portal Hypertension in Cirrhosis: Development and Validation of a Superior Non-Invasive Diagnostic Model

**DOI:** 10.5152/tjg.2025.25155

**Published:** 2025-10-10

**Authors:** Han Hu, Wei Lai, Tiantian Zhou, Jie Zhu, Huadong Yan

**Affiliations:** 1 Zhejiang Chinese Medical University, Zhejiang, China; 2 Department of Infectious Diseases, Affiliated Shulan (Hangzhou) Hospital, Shulan International Medical College, Zhejiang Shuren University, Zhejiang, China

**Keywords:** Portal hypertension, Liver cirrhosis, Indocyanine green

## Abstract

**Background/Aims:**

To assess the correlation between indocyanine green retention rate at 15 minutes (ICG-R15), liver stiffness measurement (LSM), and other clinical indicators in cirrhotic patients, using hepatic venous pressure gradient (HVPG) as a reference and to evaluate the predictive capability of ICG-R15 for clinically significant portal hypertension (CSPH).

**Materials and Methods:**

From February 2023 to September 2024, 80 cirrhotic patients were recruited. Data collected included baseline information, laboratory results, HVPG measurements, and ICG-R15 via the ICG clearance test. Patients were classified into non-CSPH (n = 33) and CSPH (n = 47) groups based on HVPG. Pearson’s correlation analyzed relationships between HVPG, ICG-R15, LSM, and other indicators. Logistic regression was used to identify risk factors for CSPH and develop a predictive model, evaluated by receiver operating characteristic curve.

**Results:**

The CSPH patients showed lower white blood cell count, red blood cell count, hemoglobin, platelet count (PLT), and albumin, with higher total bilirubin (TBil), prothrombin time, LSM, and ICG-R15. Significant correlations were found between HVPG and ICG-R15 (*r* = 0.662) and LSM (*r* = 0.633) (both *P* < .001). The ICG-R15, LSM, PLT, and TBil were independent risk factors for CSPH. The model had an AUC of 0.947, sensitivity of 78.72%, and specificity of 96.97%.

**Conclusion:**

The ICG-R15 is a significant predictor of CSPH, and a model incorporating ICG-R15 can effectively assess disease severity and predict prognosis in cirrhotic patients.

**Main Points**

- The indocyanine green retention rate at 15 minutes (ICG-R15) significantly correlates with hepatic venous pressure gradient (HVPG) and serves as an independent predictor of clinically significant portal hypertension (CSPH) in cirrhotic patients.
- A novel predictive model integrating ICG-R15, liver stiffness measurement, platelet count, and total bilirubin achieves superior diagnostic accuracy (AUC = 0.947) for CSPH.
- The new model outperforms traditional scores (Child-Turcotte-Pugh, model for end-stage liver disease, fibrosis-4, aspartate aminotransferase-to-platelet ratio index) in diagnosing CSPH, offering a non-invasive alternative to invasive HVPG measurement.
- This study validates ICG-R15’s predictive value in a Chinese cohort, emphasizing its potential for optimizing non-invasive CSPH screening and management.

## Introduction

The terminal stage of chronic liver disease frequently manifests as liver cirrhosis. Currently, approximately 2 million people worldwide die from liver-related diseases annually, including about 1 million deaths attributed to cirrhosis.^1^ Portal hypertension (PH) represents a clinical manifestation associated with liver cirrhosis, which is marked by elevated resistance within hepatic vasculature and augmented blood flow through the portal venous system. It may precipitate various decompensation events, including hemorrhage of esophagogastric varices, hepatorenal syndrome, ascites, and hepatic encephalopathy, thereby impacting prognosis.^2^^-^^4^ The hepatic venous pressure gradient (HVPG) serves as the gold standard for assessing PH severity, reflecting crucial information about cirrhotic progression and guiding both prognosis assessment and treatment planning. Clinically, PH is confirmed at HVPG values exceeding 5 mmHg, whereas clinically significant portal hypertension (CSPH) is established when measurements reach or surpass 10 mmHg, indicating higher risks of variceal complications and decompensation.^5^^,^^6^ However, HVPG measurement is invasive, costly, technically challenging, and thus difficult to widely implement.

The indocyanine green (ICG) clearance test has gained widespread clinical acceptance for evaluating hepatic functional reserve due to its non-invasive characteristics, safety profile, reproducibility, and cost-effectiveness. Research evidence demonstrates that in compensated cirrhosis patients, ICG-R15 represents the 15-minute retention rate obtained through this diagnostic method, exhibits significant correlation with HVPG, and effectively identifies or excludes CSPH in this population.^7^ However, research exploring ICG-R15’s predictive value for CSPH in cirrhosis patients remains extremely limited globally. To further investigate the clinical utility of ICG-R15 in predicting CSPH across all cirrhosis patients, this study establishes a cirrhosis cohort based on HVPG and ICG-R15 measurements. By analyzing the relationship between ICG-R15, other non-invasive indicators, and HVPG changes in cirrhosis patients, the study aims to identify risk factors influencing CSPH, clarify the predictive value of ICG-R15 for CSPH, and develop novel models for CSPH prediction. It is hypothesized that ICG-R15, combined with liver stiffness measurement (LSM) and routine biomarkers, can accurately predict CSPH non-invasively, offering a practical alternative to HVPG.

## Materials and Methods

### General Information

Patients with liver cirrhosis admitted to the Department of Infectious Diseases at Affiliated Shulan (Hangzhou) Hospital from February 2023 to September 2024 were prospectively enrolled based on inclusion and exclusion criteria. This study was approved by the Ethics Committee of Affiliated Shulan (Hangzhou) Hospital, Shulan International Medical College, Zhejiang Shuren University, January 1, 2023 (approval name: KY2022001, date: January 1, 2023), and written informed consent was obtained from every participant. The diagnosis of liver cirrhosis was made in accordance with the guidelines.^8^ All enrolled patients signed informed consent forms. Exclusion conditions: 1. Outside the age range of 18-75 years. 2. Presence of various malignancies, such as liver malignancies. 3. Comorbid severe systemic diseases, such as severe heart failure, respiratory failure, renal failure, acute cerebral hemorrhage, or cerebral infarction. 4. HIV infection. 5. Use of non-selective beta-blockers (NSBBs) for blood pressure control prior to HVPG measurement. 6. Excluded patients on NSBBs. 7. History of splenectomy or transjugular intrahepatic portosystemic shunt, or experienced severe thrombosis of the portal vein, portosystemic shunt, or portal cavernous transformation. 8. Post-liver transplantation. 9. Porto-sinusoidal vascular disease. 10. Pregnancy. 11. Failure to measure HVPG. 12. Incomplete clinical data. The HVPG and ICG-R15 assessors were blinded to each other’s results to avoid bias.

### Measurement Indicators

Upon admission, fasting laboratory tests were performed to evaluate hematological profiles, hepatic functional status, renal performance, and the function of coagulation. The specific parameters analyzed encompass white blood cell (WBC) count, red blood cell (RBC) count, hemoglobin (Hb), platelet count (PLT), albumin (Alb), total bilirubin (TBil), aspartate aminotransferase, alanine aminotransferase, serum creatinine, and prothrombin time (PT). Utilizing these laboratory results, the Child-Turcotte-Pugh (CTP), model for end-stage liver disease (MELD), fibrosis-4 (FIB-4), and aspartate aminotransferase-to-platelet ratio Index (APRI) scores are calculated. Additionally, all enrolled patients undergo LSM, ICG-R15 testing, followed by HVPG measurement.

### Liver Stiffness Measurement

Within 48 hours before or after admission, patients underwent LSM using the FibroScan 502/630 device (ECHOSENS, France).^9^ Operators were trained and certified by ECHOSENS. The standardized operation procedure was followed, with the measurement area defined as the liver region bounded by the xiphoid process, right mid-axillary line, and lower rib margin. The measurement point was selected within the intercostal space, and at least 10 successful measurements were required for each point.

### Hepatic Venous Pressure Gradient Measurement

All patients underwent HVPG measurement under local anesthesia by experienced interventional radiologists. The procedure involved ultrasound-guided vascular access employing the Seldinger method, a 7F balloon-tipped catheter into the right hepatic vein under continuous electrocardiographic surveillance, fluoroscopy, and continuous arterial pressure measurement. After balloon inflation, 5 mL of iodine contrast agent was injected to confirm complete occlusion and the absence of shunts. Triplicate measurements were obtained for both occluded and free hepatic venous pressures, and the stable difference between the 2 was recorded as HVPG. Permanent records were obtained using a multichannel recorder.

### Indocyanine Green Retention Rate at 15-Minutes

The ICG elimination assessment was performed through pulse spectrophotometric method. All patients were tested in the morning after fasting, using the DDG-3300K analyzer (Nihon Kohden, Japan). The operator measured the patient’s fasting peripheral venous Hb level, along with height and weight, and input these values into the liver reserve function analyzer. The ICG diagnostic solution was produced at 5 mg/mL through dilution of 0.5 mg/kg of ICG in 5 mL of sterile aqueous solvent. This freshly prepared contrast medium was subsequently administered via rapid intravenous bolus injection through the median cubital venous access, with the infusion completed within 5-10 seconds. The system calculated ICG-R15 after 15 minutes.

### Statistical Analysis

Data analysis was conducted utilizing SPSS 23.0 (IBM SPSS Corp.; Armonk, NY, USA). Data that conformed to a normal distribution are presented as mean ± standard deviation (x̄ ± s), and comparisons between 2 groups were conducted using the *t*-test. Non-normally distributed data are expressed as median (interquartile range), and comparisons between 2 groups were conducted using the rank-sum test. Categorical data are expressed as counts (%), and comparisons between 2 groups were conducted using the *χ*^2^ test or Fisher’s exact test. Pearson correlation coefficients were employed to examine associations among clinical parameters. To ascertain the risk elements associated with CSPH, both univariate and multivariate logistic regression models were implemented. The diagnostic performance of clinical markers was quantified through receiver operating characteristic curve (ROC) analysis, with the calculated area under the curve (AUROC) serving as the primary metric for predictive accuracy.* P* < .05 was established as the criterion for statistical significance.

## Results

### Baseline Characteristics

The study cohort comprised 105 cirrhotic patients initially evaluated for eligibility, with 25 individuals excluded based on predefined exclusion parameters. Ultimately, 80 patients with liver cirrhosis were included, consisting of 56 male subjects (70.0%) and 24 female subjects (30.0%), with a median age of 54 years (interquartile range: 14.8 years). Among them, 19 patients (23.8%) had diabetes, and 33 patients (41.3%) had a history of decompensation. Regarding disease etiology, hepatitis B virus infection represented the predominant cause (52 cases), subsequently succeeded by alcohol-related liver damage (14 cases), including 4 cases of combined hepatitis B and alcoholic cirrhosis. Other etiologies accounted for 18 cases. A total of 47 patients (58.8%) developed CSPH.

Further grouping based on CSPH status revealed 47 cases with CSPH and 33 cases without CSPH, with comparative analyses presented in Table 1. The CSPH-positive cohort demonstrated significantly elevated rates of diabetic comorbidity and prior decompensation episodes relative to the CSPH-negative group (*P* < .05). Age, gender composition, body mass index, and underlying etiologies showed no statistically significant intergroup differences (*P* > .05). Laboratory parameter analysis revealed marked reductions in WBC, RBC, Hb, PLT, and Alb levels in the CSPH-positive group, accompanied by substantial elevations in TBil, PT, LSM, and ICG-R15 (*P* < .05). There were no significant differences in MELD score between groups (*P* > .05), and the CSPH-positive cohort exhibited significantly higher values in CTP, FIB-4 index, and APRI (*P* < .05).

### Correlation Between Hepatic Venous Pressure Gradient and Related Indicators

**Figure 1.**
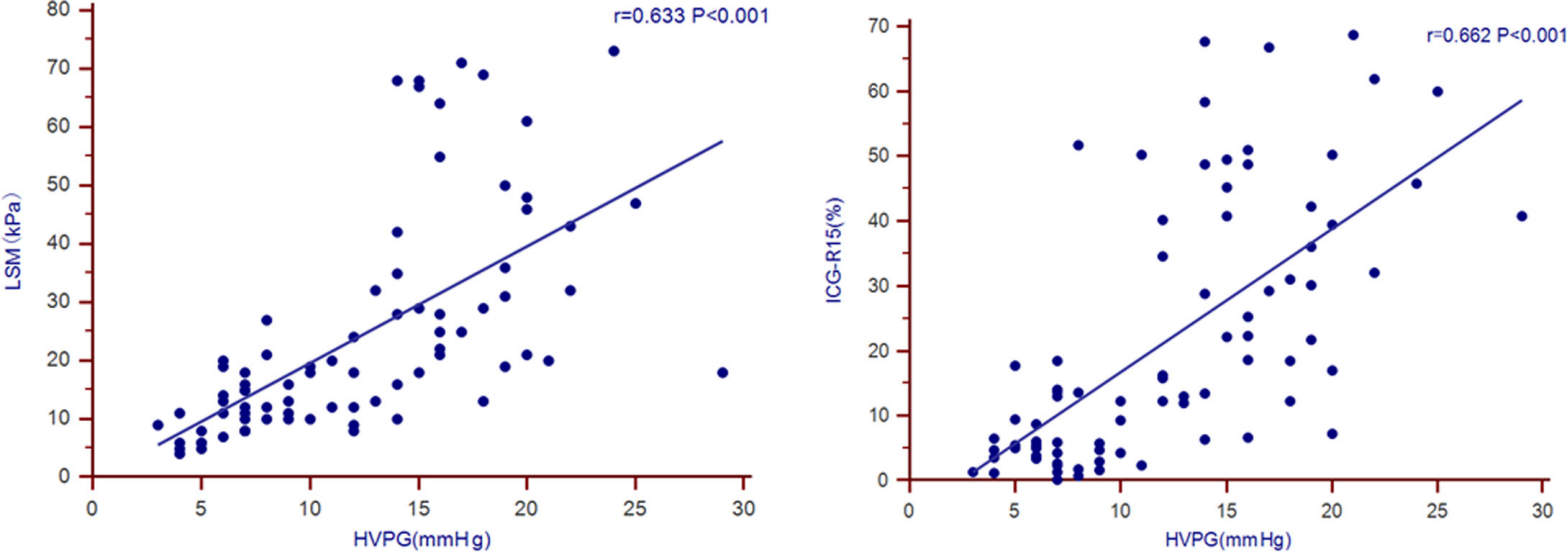
Correlation analysis of HVPG with ICG-R15 and LSM. HVPG, hepatic venous pressure gradient; ICG-R15, indocyanine green retention rate at 15 minutes; LSM, liver stiffness measurement.

Pearson correlation analysis was performed between HVPG and WBC, RBC, PLT, Alb, LSM, ICG-R15, CTP score, and FIB-4 score, as shown in Table 2. The HVPG demonstrated strong statistical associations with ICG-R15 and LSM, exhibiting Pearson’s correlation coefficients calculated at 0.662 and 0.633, respectively (both* P* < .001), as visually represented in Figure 1.

### Risk Factors for Clinically Significant Portal Hypertension in Liver Cirrhosis

Univariate logistic regression analysis identified diabetes, previous decompensation, WBC, RBC, Hb, PLT, Alb, TBil, PT, ICG-R15, and LSM as significant predictors for CSPH in cirrhotic patients, as detailed in Table 3. Subsequent multivariate analysis identified 4 independent predictive variables for CSPH development: ICG-R15, LSM, PLT, and TBil in cirrhosis patients, with detailed data presented in Table 4.

### Development of a New Model for Predicting Clinically Significant Portal Hypertension in Patients with Liver Cirrhosis

**Figure 2.**
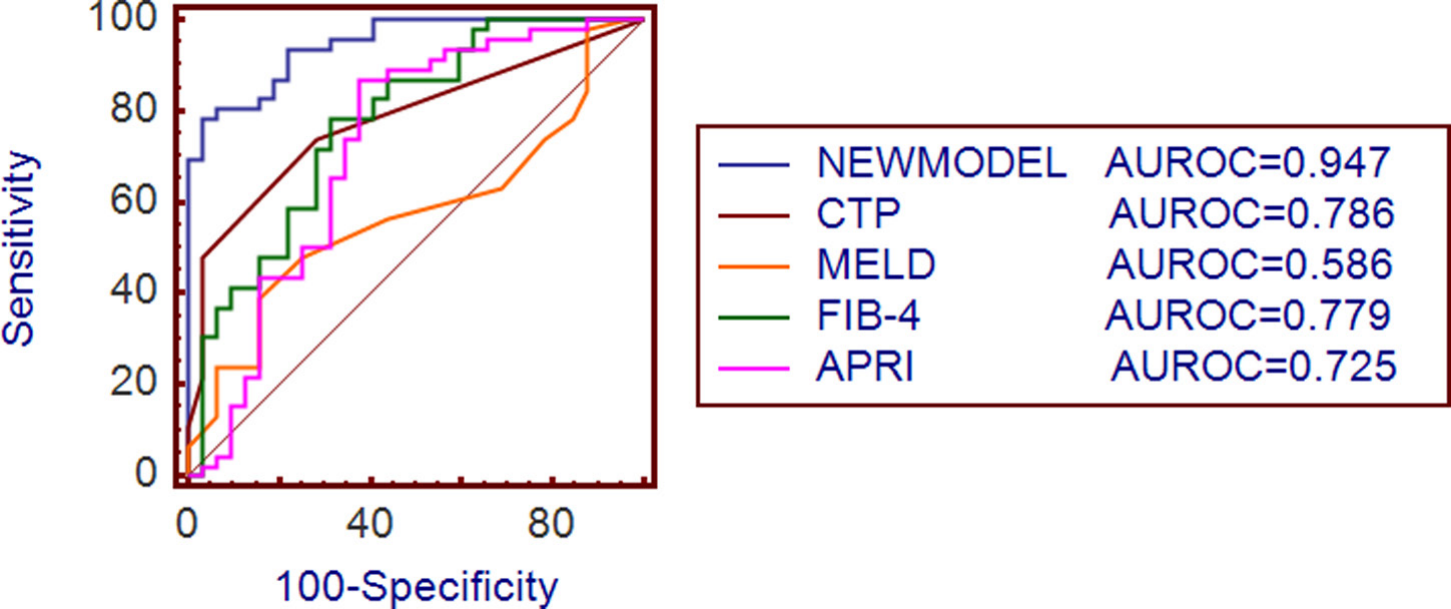
ROC curve of different clinical scores and new model in predicting CSPH in patients with liver cirrhosis. APRI, aspartate aminotransferase-to-platelet ratio index; CTP, Child-Turcotte-Pugh; FIB-4, fibrosis-4; MELD, model for end-stage liver disease.

Based on multivariate logistic regression analysis, ICG-R15, LSM, PLT, and TBil were necessary variables for inclusion in the model, resulting in the equation: Y = 0.142 × ICG − R15 + 0.144 × LSM − 0.022 × PLT − 0.113 × TBil − 0.043. This equation was utilized to develop a novel predictive model. The ROC curves were thus generated for both the newly proposed model and the 4 aforementioned prognostic scores (CTP, MELD, FIB-4, and APRI). The new model had a cutoff value of 1.13, with a sensitivity of 78.72% and a specificity of 96.97%. The area under the curve (AUC) for the new model was determined to be 0.947 (95% CI: 0.872-0.985), which surpassed the AUC values of the CTP, MELD, FIB-4, and APRI (AUC = 0.786, 0.586, 0.779, 0.725, respectively), as depicted in Figure 2. It was confirmed by Delong’s test that the AUC value predicted by the new model was significantly higher than that of the CTP, MELD, FIB-4, and APRI (*Z* = 3.506, 5.221, 3.562, 3.810, *P <* .001).

## Discussion

In this study, 80 patients were enrolled, among whom 47 (58.8%) developed CSPH, for whom patients were treated with carvedilol to reduce portal pressure, and the treatment was well tolerated. The statistical results revealed the presence of significant correlations between HVPG and both ICG-R15 and LSM, with Pearson’s correlation coefficients measuring 0.662 and 0.633, respectively (both *P* < .001). Univariate analysis revealed that diabetes, previous decompensation, WBC, RBC, Hb, PLT, Alb, TBil, PT, ICG-R15, and LSM were all factors associated with CSPH. Multivariate analysis identified ICG-R15, LSM, PLT, and TBil as independent factors influencing CSPH. The new model established based on multivariate analysis had an AUC of 0.947, outperforming the CTP (AUC = 0.786), MELD (AUC = 0.586), FIB-4 (AUC = 0.779), and APRI (AUC = 0.725) scores.

The CSPH represents a crucial target for intervention in patients suffering from liver cirrhosis and PH. Currently, the definitive method for diagnosing CSPH is HVPG measurement.^10^ However, the invasiveness, technical complexity, and high cost of HVPG measurement limit its widespread use in clinical practice. Thus, it is particularly imperative to develop non-invasive models to identify CSPH. The ICG, a biocompatible synthetic tricarbocyanine dye, demonstrates unique pharmacokinetic properties including exclusive hepatic uptake, biliary excretion without enterohepatic recirculation, and absence of extrahepatic metabolism. These undergo no extraintestinal metabolism or excretion, and its excretion rate depends on hepatic functional capacity and perfusion, establishing ICG as a reliable marker for assessing liver reserve function.^11^ Existing studies have confirmed that the ICG clearance test is useful in monitoring hepatic hemodynamic alterations.^12^^,^^13^ As liver cirrhosis progresses, increased intrahepatic vascular resistance and portal blood flow contribute to elevated portal pressure.^14^ Therefore, it is common for patients with PH to exhibit increased ICG-R15 as HVPG rises. The ICG-R15 significantly correlated with HVPG and was an independent predictor of CSPH. Thus, it is believed that ICG-R15 has promising potential for identifying CSPH. Its non-invasive, rapid, low-cost, and easy-to-use characteristics make it suitable for screening large numbers of patients for PH in clinical settings. Additionally, TBil and PLT, as conventional markers of PH in liver cirrhosis, are often elevated and reduced, respectively, in CSPH patients due to hepatocyte damage and hypersplenism. Currently, TBil and PLT are used in various prognostic models for liver cirrhosis, such as CTP, MELD, FIB-4, and APRI. The LSM is also an independent risk factor for identifying CSPH. It seems that MELD score was not associated with CSPH. The possible reasons for this analysis might be that MELD and CSPH assess distinct aspects of liver disease (survival vs. hemodynamics). The cohort had a median MELD score of 5.0 (IQR: 4.2), with 90% of patients scoring <10, a narrow MELD range, and low decompensation rates in the cohort limited statistical power. There were fewer decompensation events in the patient group, which affected the correlation of the MELD score. Compared to HVPG measurement, LSM is non-invasive, repeatable, cost-effective, and easy to perform. The findings align with previous studies demonstrating the utility of non-invasive markers in assessing PH. For instance, Lantinga et al^15^ established spleen stiffness measurement using elastography techniques, which shows improved accuracy for CSPH prediction when combined with LSM. However, the study extends these findings by incorporating ICG-R15, which directly reflects hepatic perfusion, thereby enhancing diagnostic accuracy. Unlike the Baveno VII criteria (which uses LSM and platelet count to rule out high-risk varices), the model incorporates ICG-R15 to directly assess hepatic functional reserve and perfusion. This may improve risk stratification for CSPH in patients where Baveno VII criteria are indeterminate, particularly those with discordant LSM and platelet values. In resource-limited settings, the model could serve as a second-line tool after Baveno VII to reduce unnecessary HVPG referrals.

In conclusion, ICG-R15 displays a substantial correlation with HVPG and is an independent risk factor for CSPH. The new model combining ICG-R15, LSM, PLT, and TBil can effectively identify CSPH in cirrhosis patients, facilitating timely intervention, thereby optimizing patient prognoses. The study has limitations. First, the single-center cohort may introduce selection bias. Second, the majority of cases were attributed to HBV and alcoholic cirrhosis, limiting the generalizability of the findings to other etiologies such as non-alcoholic steatohepatitis or autoimmune liver diseases. Additionally, in patients with severe cholestasis or acute hepatic decompensation, ICG-R15 measurements may be confounded by altered bilirubin metabolism or hepatic blood flow. Future multicenter studies encompassing diverse etiologies and disease severities are needed to validate the model’s universal applicability.

## Figures and Tables

**Table 1.** Characteristics of the Included Patients

Parameter	Overall Cohort (n = 80)	Non-CSPH Patients (n = 33)	CSPH Patients (n = 47)	Statistical Value	*P*
Age (years)	54.0 (14.8)	51.0 (16.5)	57.0 (14.0)	*Z* = −1.121	.262
Male (%)	56 (70.0)	24 (72.7)	32 (68.1)	*χ*^2^= 0.199	.656
Diabetes (%)	19 (23.8)	4 (12.1)	15 (31.9)	*χ*^2^= 4.194	.041
BMI (kg/m^2^)	24.9 (4.9)	24.5 (5.6)	25.3 (5.1)	*Z* = -0.45	.653
Previous decompensation (%)				*χ*^2^= 28.701	<.001
Ascites	22 (27.5)	1 (3.0)	21 (44.7)		
Variceal bleeding	7 (8.8)	1 (3.0)	6 (12.8)		
Hepatic encephalopathy	4 (5.0)	0 (0.0)	4 (8.5)		
Etiology					
HBV (%)	52 (65.0)	24 (72.7)	28 (59.6)	*χ*^2^= 1.474	.225
Alcohol (%)	14 (17.5)	4 (12.1)	10 (21.3)	*χ*^2^= 1.123	.289
Others (%)	18 (22.5)	7 (21.2)	11 (23.4)	*χ*^2^= 0.053	.817
Lab tests					
WBC (10^9^/L)	3.8 ± 1.6	4.3 ± 1.6	3.4 ± 1.6	*t* = 2.381	.02
RBC (10^9^/L)	4.1 ± 0.8	4.7 ± 0.5	3.8 ± 0.8	*t* = 5.826	<.001
Hemoglobin (g/L)	124.3 ± 27.1	141.3 ± 18.2	112.4 ± 26.1	*t* = 5.839	<.001
PLT (10^9^/L)	84.5 (64.5)	113.0 (79.0)	72.0 (46.0)	*Z* = −3.758	<.001
ALB (g/L)	38.2 ± 6.3	42.6 ± 4.0	35.0 ± 5.7	*t* = 7.061	<.001
Total bilirubin (µmol/L)	19.0 (15.5)	18.0 (8.0)	21.0 (18.0)	*Z* = −2.532	.011
AST (U/L)	32.5 (17.8)	26.0 (16.0)	34.0 (18.0)	*Z* = −1.614	.107
ALT (U/L)	23.0 (17.5)	23.0 (37.5)	24.0 (13.0)	*Z* = −0.396	.692
Creatinine (µmol/L)	68.3 ± 17.9	72.6 ± 16.0	65.3 ± 18.6	*t* = 1.835	.07
PT (s)	12.8 (2.4)	11.6 (1.0)	13.9 (2.7)	*Z* = −6.14	<.001
ICG-R15 (%)	13.7 (33.6)	5.0 (6.6)	30.1 (35.3)	*Z* = −6.045	<.001
LSM (kPa)	18.0 (20.2)	10.8 (7.1)	27.6 (28.5)	*Z* = −5.816	<.001
HVPG (mmHg)	12.0 (9.0)	7.0 (2.5)	16.0 (6.0)	*Z* = −7.596	<.001
CTP score, points	6.0 (2.0)	5.0 (1.0)	6.0 (3.0)	*Z* = −4.637	<.001
MELD score, points	5.0 ± 4.2	4.0 ± 3.7	5.6 ± 4.4	*t* = −1.651	.103
FIB-4 score, points	4.2 (4.4)	2.7 (3.0)	5.6 (4.2)	*Z* = −4.334	<.001
APRI score, points	1.1 (1.2)	0.7 (1.0)	1.3 (1.0)	*Z* = −3.533	<.001

ALB, albumin; ALT, alanine aminotransferase; APRI, aspartate aminotransferase-to-platelet ratio index; AST, aspartate aminotransferase; BMI, body mass index; CTP, Child-Turcotte-Pugh; FIB-4, fibrosis-4; HBV, hepatitis B virus; HVPG, hepatic venous pressure gradient; ICG-R15, indocyanine green retention rate at 15 minutes; LSM, liver stiffness measurement; MELD, model for end-stage liver disease; PLT, platelet count; PT, prothrombin time; RBC, red blood cell count; WBC, white blood cell count.

**Table 2.** Correlation Analysis Results of HVPG with ICG-R15 and Related Indicators in Overall Patients

Parameter	*r*-	*P*
WBC (10^9^/L)	−0.289	.009
RBC (10^9^/L)	−0.545	<.001
Hemoglobin (g/L)	−0.538	<.001
PLT (10^9^/L)	−0.396	<.001
ALB (g/L)	−0.592	<.001
Total bilirubin (µmol/L)	0.348	.002
PT (s)	0.518	<.001
ICG-R15 (%)	0.662	<.001
LSM (kPa)	0.633	<.001
CTP score, points	0.490	<.001
FIB-4 score, points	0.340	.002

ALB, albumin; CTP, Child-Turcotte-Pugh; FIB-4, fibrosis-4; ICG-R15, indocyanine green retention rate at 15 minutes; LSM, liver stiffness measurement; PLT, platelet count; PT, prothrombin time; WBC, white blood cell count.

**Table 3.** Logistic Univariate Regression Analysis Results of Clinical Significant Portal Hypertension in Overall Patients

Parameter	*B*-value	*Wald*-value	OR, 95% CI	*P*
Age (years)	0.033	1.706	1.033 (0.984-1.085)	.192
Male (%)	0.223	0.199	1.25 (0.469-3.335)	.656
Diabetes (%)	1.223	3.913	3.398 (1.011-11.42)	.048
BMI (kg/m^2^)	0.001	0.001	1.001 (0.915-1.096)	.977
Previous decompensation (%)	3.402	18.461	30.031 (6.361-141.772)	<.001
Etiology				
HBV (%)	−0.593	1.459	0.553 (0.211-1.447)	.227
Alcohol (%)	0.673	1.100	1.959 (0.557-6.889)	.294
Others (%)	0.127	0.053	1.135 (0.388-3.32)	.817
Lab tests				
WBC (10^9^/L)	−0.348	5.100	0.706 (0.522-0.955)	.024
RBC (10^9^/L)	−2.025	15.713	0.132 (0.048-0.359)	<.001
Hemoglobin (g/L)	−0.057	16.705	0.945 (0.92-0.971)	<.001
PLT (10^9^/L)	−0.023	12.84	0.977 (0.965-0.99)	<.001
ALB (g/L)	−0.302	19.583	0.739 (0.647-0.845)	<.001
Total bilirubin (µmol/L)	0.043	4.192	1.043 (1.002-1.087)	.041
AST (U/L)	−0.003	0.613	0.997 (0.991-1.004)	.434
ALT (U/L)	−0.013	1.665	0.987 (0.967-1.007)	.197
Creatinine (µmol/L)	−0.024	3.168	0.977 (0.951-1.002)	.075
PT (s)	1.437	18.260	4.207 (2.177-8.132)	<.001
ICG-R15 (%)	0.140	15.283	1.151 (1.072-1.234)	<.001
LSM (kPa)	0.212	15.397	1.236 (1.112-1.374)	<.001
CTP score (points)	1.103	11.594	3.013 (1.597-5.686)	.001
MELD score (points)	0.095	2.599	1.1 (0.98-1.235)	.107
FIB-4 score (points)	0.271	7.642	1.311 (1.082-1.589)	.006
APRI score (points)	0.029	0.083	1.03 (0.845-1.255)	.773

ALB, albumin; ALT, alanine aminotransferase; APRI, aspartate aminotransferase-to-platelet ratio index; AST, aspartate aminotransferase; BMI, body mass index; CTP, Child-Turcotte-Pugh; FIB-4, fibrosis-4; HBV, hepatitis B virus; ICG-R15, indocyanine green retention rate at 15 minutes; LSM, liver stiffness measurement; MELD, model for end-stage liver disease; PLT, platelet count; PT, prothrombin time; RBC, red blood cell count; WBC, white blood cell count.

**Table 4.** Logistic Multivariate Regression Analysis Results of Clinical Significant Portal Hypertension in Overall Patients

Parameter	*B*-value	*Wald*-value	OR, 95% CI	*P*
ICG-R15 (%)	0.142	6.054	1.152 (1.029-1.29)	.014
LSM (kPa)	0.144	5.215	1.155 (1.021-1.307)	.022
PLT (10^9^/L)	−0.022	5.018	0.978 (0.959-0.997)	.025
Total bilirubin (µmol/L)	−0.113	4.24	0.893 (0.802-0.995)	.039
Constant	−0.043	0.001	0.958	.972

ICG-R15, indocyanine green retention rate at 15 minutes; LSM, liver stiffness measurement; PLT, platelet count.

## Data Availability

The data that support the findings of this study are available from the corresponding author, upon reasonable request.
